# Shared exposure liability of type 2 diabetes and other chronic conditions in the UK Biobank

**DOI:** 10.1007/s00592-022-01864-5

**Published:** 2022-03-29

**Authors:** Yixuan He, Chirag J. Patel

**Affiliations:** 1 Program in Bioinformatics and Integrative Genomics, Harvard Medical School, 10 Shattuck St, Boston, MA 02215 USA; 2 Department of Biomedical Informatics, Harvard Medical School, 10 Shattuck St, Boston, MA USA

**Keywords:** Polyexposure risk score, Polygenic risk score, Exposome

## Abstract

**Aims:**

To investigate whether the cumulative exposure risks of incident T2D are shared with other common chronic diseases.

**Research design and methods:**

We first establish and report the cross-sectional prevalence, cross-sectional co-prevalence, and incidence of seven T2D-associated chronic diseases [hypertension, atrial fibrillation, coronary artery disease, obesity, chronic obstructive pulmonary disease (COPD), and chronic kidney and liver diseases] in the UK Biobank. We use published weights of genetic variants and exposure variables to derive the T2D polygenic (PGS) and polyexposure (PXS) risk scores and test their associations to incident diseases.

**Results:**

PXS was associated with higher levels of clinical risk factors including BMI, systolic blood pressure, blood glucose, triglycerides, and HbA1c in individuals without overt or diagnosed T2D. In addition to predicting incident T2D, PXS and PGS were significantly and positively associated with the incidence of all 7 other chronic diseases. There were 4% and 8% of individuals in the bottom deciles of PXS and PGS, respectively, who were prediabetic at baseline but had low risks of T2D and other chronic diseases. Compared to the remaining population, individuals in the top deciles of PGS and PXS had particularly high risks of developing chronic diseases. For instance, the hazard ratio of COPD and obesity for individuals in the top T2D PXS deciles was 2.82 (95% CI 2.39–3.35, *P* = 4.00 × 10^−33^) and 2.54 (95% CI 2.24–2.87, *P* = 9.86 × 10^−50^), respectively, compared to the remaining population. We also found that PXS and PGS were both significantly (*P* < 0.0001) and positively associated with the total number of incident diseases.

**Conclusions:**

T2D shares polyexposure risks with other common chronic diseases. Individuals with an elevated genetic and non-genetic risk of T2D also have high risks of cardiovascular, liver, lung, and kidney diseases.

**Supplementary Information:**

The online version contains supplementary material available at 10.1007/s00592-022-01864-5.

## Introduction

Type 2 diabetes (T2D) is a progressive chronic disease that has become one of the fastest growing health challenges in the world. A key feature of T2D is that it is often accompanied by or is an antecedent to several other chronic diseases, including cardiovascular diseases, liver, and kidney diseases. Recent reports have shown that 75–90% of people with T2D have at least one other condition [1, 2], which can affect the prognosis, survival, and quality of life of these patients [3, 4]. Upstream factors, such as BMI and prediabetes, confer later risk of T2D and other chronic diseases [5, 6]. However, not everyone who has these risk factors progresses to diabetes or other diseases [7, 8]. Here, we ask: do environmental and genetic factors that predispose individuals to T2D also implicate elevated risk of other associated chronic diseases, even in individuals who do not have T2D at baseline?

There are some known shared risk genetic alleles and environmental risk factors between T2D and other chronic diseases. For example, in genetics, Phenome-Wide Association Studies (PheWASs) have found common associations between T2D-associated variants and multiple other chronic diseases, including obesity, hypertension, and atrial fibrillation [9]. However, these single genetic variants alone cannot fully explain the co-occurrence and longitudinal risk of T2D and its related diseases. Similarly, while evidence is accumulating for individual factors such as smoking and physical activity in their roles in multiple chronic phenotypes, most investigations do not study them simultaneously and have investigated only a few exposures at a time. It remains unknown whether the combined environmental risk factors for T2D can also predict the risk of onset of other associated diseases [10].

We previously found evidence of additive exposure risks from several non-genetic factors, such as alcohol intake, past tobacco usage, and diet upstream of T2D diagnosis which we termed the polyexposure risk score (PXS) [11]. Here, we sought to investigate whether cumulative exposure risks of T2D also pose risks for its related chronic diseases, and we compared this risk to that imparted by static genetic variants.

Specifically, we aim to investigate the shared polygenic and polyexposure risks of T2D and seven related chronic diseases in the UK Biobank, a prospective convenience cohort with over half a million participants recruited from the UK with linked healthcare records and self-reported questionnaire data [12]. We demonstrate that individuals with elevated T2D polygenic and polyexposure risk scores have evidence of “subclinical” T2D—e.g., non-overt prediabetes—as well as its associated risk of chronic diseases.

## Results

### Prevalence and co-prevalence of T2D-associated diseases in the UK Biobank

We identified 21,935 individuals from the UK Biobank diagnosed with T2D from 1977 to 2019, of whom 13,567 were men and 8,368 were women, with a median year of birth of 1946. We estimated the cross-sectional prevalences of seven other chronic diseases in individuals with T2D: hypertension (78.24%), coronary artery disease (18.93%), obesity (17.67%), atrial fibrillation (13.68%), chronic obstructive pulmonary disease (COPD) (11.30%), chronic kidney disease (11.52%), and chronic liver disease (4.85%) (Table 1). Diseases were more prevalent in males and older individuals, except obesity and chronic liver disease, which were more prevalent in females and younger individuals. The prevalence of all diseases in age and sex subgroups of individuals with diabetes is shown in Supplementary Table 1.

**Table 1:** Prevalence and odds ratio (OR) of diseases in individuals with and without T2D

Disease	T2D individuals				Sex- and age-matched non-T2D individuals		T2D versus matched non-T2D individuals	
	Prevalence (%)		Age at T2D diagnosis (median)	Age at disease diagnosis (median)	Prevalence (%)		OR	CI
HT	17,163	78.24	63	54	8238	37.56	5.13	4.66–5.65
AF	3000	13.68	64	66	1397	6.37	6.53	5.54–7.73
CAD	4152	18.93	63	58	1416	6.46	3.89	3.56–4.24
Obesity	3876	17.67	60	63	701	3.20	2.33	2.18–2.49
CLD	1064	4.85	60	63	170	0.78	3.38	3.17–3.61
CKD	2526	11.52	63	67	543	2.48	5.98	5.73–6.24
COPD	2478	11.30	63	62	696	3.17	6.50	5.98–7.07

All T2D versus matched non-T2D comparisons were significant (*P* < 0.0001)

*AF* Atrial fibrillation, *HT* Hypertension, *CAD* Coronary artery disease, *CKD* Chronic kidney disease, *COPD* Chronic obstructive pulmonary disease, *CLD* Chronic liver disease, *CI* Confidence interval

In the sex- and age-matched group of individuals without diabetes, the prevalence of all diseases was significantly lower. Hypertension, atrial fibrillation, and coronary artery disease more than halved to 37.56%, 6.37%, and 6.46%, respectively, in participants without diabetes. Obesity decreased over fivefold to 3.2%, and chronic liver disease was almost non-existent (0.78%) in the individuals without diabetes. The prevalence of diseases in age and sex subgroups for individuals without diabetes is shown in Supplementary Table 2. Individuals with T2D have a greater than fivefold cross-sectional odds of having atrial fibrillation, chronic obstructive pulmonary disease, coronary artery disease, and chronic kidney disease than individuals without T2D (Table 1, Supplementary Fig. 1a).

Of the participants with diabetes, 3,294 (15.02%) were not diagnosed with any other disease during the time of surveillance, 9,018 (41.11%) had one other disease, 5,554 (25.32%) had two other diseases, 2,595 (11.83%) had three other diseases, and 1,474 (6.72%) had four or more other diseases (Supplementary Fig. 1b, Supplementary Tables 3). The highest co-prevalence was the combination of hypertension and coronary artery disease (6.84%) (Supplementary Table 4). In individuals with T2D, the median year of birth of individuals without a diagnosis of any other disease was 1948 (IQR = 11 years), and that of individuals with only one other disease and that of individuals with two or more other diseases are 1947 (IQR = 10 years) and 1946 (IQR = 11 years), respectively.

### T2D polygenic and polyexposure risk scores

We calculated pre-validated T2D polyexposure risk scores (PXS) and polygenic risk scores (PGS) for 68,132 participants [median (IQR) year of birth, 1951 [13] years; 35,580 (52.22%) females] from the UK Biobank who did not have T2D at baseline. PGS is derived from the weighted sum of over 6 million genetic variants. PXS measures the non-genetic exposure risk of an individual based on the weighted sum of 12 environmental exposures (out of 111 factors), including alcohol intake, comparative body size at age 10 years, major dietary changes in the past 5 years, household income, insomnia, snoring, milk type used (skim, whole, etc.), dietary restriction (eggs, dairy, wheat, etc.), spread type used (butter, etc.), and tea intake per day. The weights of T2D PGS and PXS are previously published elsewhere [11, 13] (Methods). Individuals who later developed T2D [*N* = 1183, median (IQR) time of 5.4 (2.93) years after assessment] had higher PXS and PGS at baseline, as well as abnormal levels of all cardiometabolic markers (Supplementary Table 5). They were also older, on average, by 4 years and were more likely to be male. 35.87% of individuals with incident T2D had a family history of T2D, while only 19.69% of individuals who do not develop T2D had a family history of T2D. Sex- and age-stratified analysis and additional summary data on cardiometabolic baseline characteristics are presented in Supplementary Table 5.

### PXS identifies individuals with an elevated clinical risk of T2D

**Fig. 1:**
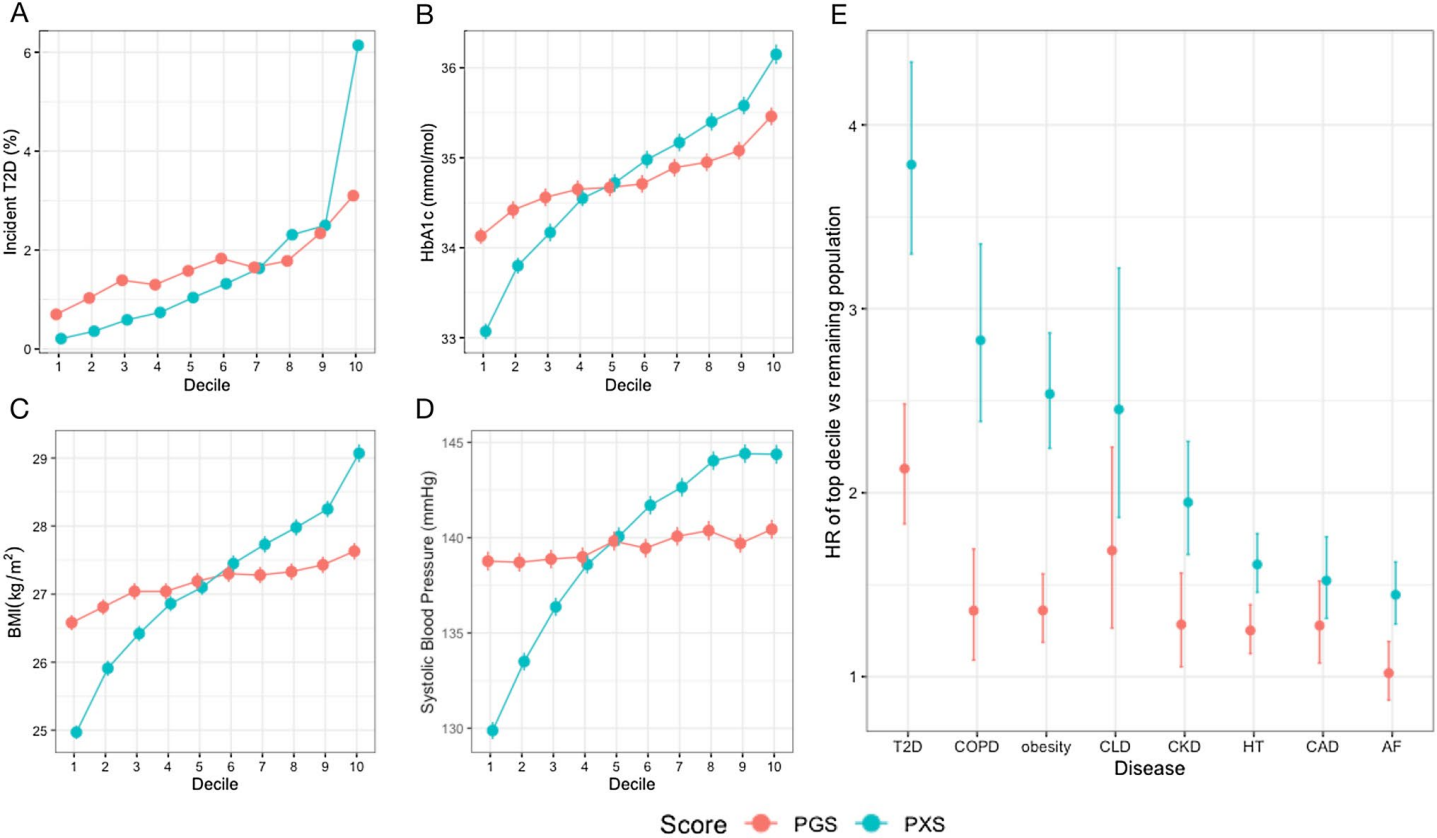
Characteristics of polygenic risk score (PGS) or polyexposure risk score (PXS) deciles in baseline T2D-free individuals for **a** number of incident T2D, **b** baseline hemoglobin A1C (HbA1C) levels, **c** baseline body mass index (BMI), and **d** baseline systolic blood pressure. Error bars represent 95% confidence intervals. *E* Hazard ratio (HR) of each disease for the top decile of polygenic risk score (PGS) or polyexposure risk score (PXS) compared to the remaining population after adjusting for age, sex, assessment center, and genetic principal components. *CLD* Chronic liver disease, *CAD* Coronary artery disease, *AF* Atrial fibrillation, *HT* Hypertension, *CKD* Chronic kidney disease, *COPD* Chronic obstructive pulmonary disease. Error bars represent 95% confidence intervals

Individuals without T2D at baseline were binned into deciles based on their PXS and PGS. Higher deciles of PXS and PGS were associated with elevated levels of clinical risk factors for T2D, including BMI, systolic blood pressure, blood glucose, triglycerides, and HbA1c (Fig. 1a–d, Table 2). We also observed that more individuals in the top deciles of PXS and PGS were prediabetic (HbA1c between 39 and 48 mmol/mol) at baseline: 23% and 17% of individuals in the top deciles of PXS and PGS, respectively, were prediabetic, while 8% and 4% of individuals in the bottom deciles of PXS and PGS, respectively, were prediabetic. Individuals with higher PXS also had higher future T2D incidence and shorter time to T2D onset (Table 2). PXS achieved greater discrimination of all variables compared to PGS. For example, 6.38% of individuals in the top decile of PXS had incident T2D in future versus 0.21% of individuals in the bottom decile of PXS had incident T2D (median 5.2 vs. 6.3 years after the first assessment, respectively). In comparison, 3.16% of individuals in the bottom decile of PGS had a future incidence of T2D versus 0.76% of individuals in the bottom decile of PGS had incident T2D (Fig. 1a). The mean BMI for individuals in the top versus bottom deciles of PXS was 29.09 and 24.98, respectively. The mean BMI for individuals in the top versus bottom deciles of PGS was 27.65 and 26.59, respectively (Fig. 1c). PXS alone was also predictive of future T2D incidence (AUC = 0.747, 95% CI 0.733–0.761) (Supplementary Fig. 2). We estimated that the sensitivity was 0.665 and the specificity was 0.697 at the AUC inflection point.

**Table 2:** Participant characteristics of each risk score decile

	Risk score deciles										Linear regression results	
	*D*1	*D*2	*D*3	*D*4	*D*5	*D*6	*D*7	*D*8	*D*9	*D*10	*β*	*P* value
*Polygenic risk score *(*PGS*)												
*N* (66,039)	6598	6613	6635	6611	6582	6624	6619	6587	6585	6585		
Time to T2D onset (yr), mean	5.85	5.57	6.16	5.51	4.89	5.38	5.82	5.23	5.49	5.16	− 0.024	0.248
Age, median ± sd	57 (8.05)	57 (8.08)	57 (8.03)	57 (7.92)	57 (8)	57 (8.08)	57 (8.05)	56 (8.06)	56 (8.07)	56 (8.02)	0.042	8.82 × 10^−05^
Systolic blood pressure (mmHg), mean ± sd	138.77 (0)	138.71 (0)	138.88 (0)	138.99 (0)	139.82 (0)	139.45 (0)	140.07 (0)	140.37 (0)	139.7 (0)	140.44 (0)	0.2	1.61 × 10^−14^
BMI, mean ± sd	26.58 (0)	26.81 (0)	27.04 (0)	27.04 (0)	27.19 (0)	27.3 (0)	27.28 (0)	27.33 (0)	27.43 (0)	27.63 (0)	0.099	8.36 × 10^−62^
Blood glucose (mmol/L), mean ± sd	4.92 (0)	4.93 (0)	4.94 (0)	4.95 (0)	4.98 (0)	4.97 (0)	4.99 (0)	5 (0)	5 (0)	5.04 (0)	0.012	1.15 × 10^−41^
HDL cholesterol (mmol/L), mean ± sd	1.5 (0)	1.49 (0)	1.48 (0)	1.47 (0)	1.47 (0)	1.46 (0)	1.46 (0)	1.46 (0)	1.44 (0)	1.43 (0)	− 0.007	4.24 × 10^−46^
Triglycerides (mmol/L), mean ± sd	1.59 (0)	1.64 (0)	1.66 (0)	1.7 (0)	1.7 (0)	1.71 (0)	1.71 (0)	1.76 (0)	1.76 (0)	1.81 (0)	0.02	2.86 × 10^−53^
HbA1c (mmol/mol), mean ± sd	34.13 (0)	34.42 (0)	34.56 (0)	34.65 (0)	34.67 (0)	34.71 (0)	34.89 (0)	34.95 (0)	35.08 (0)	35.46 (0)	0.117	3.05 × 10^−124^
PXS decile, median ± sd	5 (2.88)	5 (2.87)	5 (2.85)	6 (2.87)	6 (2.85)	5 (2.89)	5 (2.87)	5 (2.87)	5 (2.86)	6 (2.89)	–	–
PRS decile, median ± sd	1 (0)	2 (0)	3 (0)	4 (0)	5 (0)	6 (0)	7 (0)	8 (0)	9 (0)	10 (0)	–	–
Males (%)	3191 (48.36)	3136 (47.42)	3213 (48.43)	3170 (47.95)	3261 (49.54)	3048 (46.01)	3098 (46.8)	3114 (47.27)	3180 (48.29)	3158 (47.96)	–	–
Family history of T2D (%)	938 (14.22)	1111 (16.8)	1124 (16.94)	1170 (17.7)	1261 (19.16)	1385 (20.91)	1443 (21.8)	1425 (21.63)	1531 (23.25)	1786 (27.12)	–	–
High ASCVD risk (%)	2543 (0.7)	2489 (1.03)	2532 (1.39)	2531 (1.3)	2594 (1.58)	2507 (1.83)	2532 (1.65)	2537 (1.78)	2555 (2.34)	2503 (3.1)	–	–
Prediabetic at baseline (%)	46 (0.7)	68 (1.03)	92 (1.39)	86 (1.3)	104 (1.58)	121 (1.83)	109 (1.65)	117 (1.78)	154 (2.34)	204 (3.1)	–	–
Future incident T2D (%)	515 (7.81)	607 (9.18)	678 (10.22)	727 (11)	758 (11.52)	750 (11.32)	849 (12.83)	893 (13.56)	981 (14.9)	1137 (17.27)	–	–
*Polyexposure risk score *(*PXS*)												
*N* (66,039)	6711	6645	6643	6643	6622	6593	6609	6588	6547	6438		
Time to T2D onset (yr), mean	6.3	5.48	6.26	5.27	5.94	5.38	5.17	5.82	5.27	5.13	− 0.079	1.01 × 10^−03^
Age, median ± sd	48 (6.06)	51 (6.91)	53 (7.3)	55 (7.3)	56 (7.24)	58 (6.99)	59 (6.8)	60 (6.51)	62 (6.01)	63 (5.5)	− 1.54	0.00
Systolic blood pressure (mmHg), mean ± sd	129.88 (0)	133.5 (0)	136.37 (0)	138.6 (0)	140.06 (0)	141.7 (0)	142.65 (0)	144.04 (0)	144.41 (0)	144.38 (0)	1.59	0.00
BMI, mean ± sd	24.97 (0)	25.91 (0)	26.42 (0)	26.86 (0)	27.1 (0)	27.45 (0)	27.73 (0)	27.98 (0)	28.25 (0)	29.07 (0)	0.387	0.00
Blood glucose (mmol/L), mean ± sd	4.86 (0)	4.89 (0)	4.91 (0)	4.95 (0)	4.97 (0)	4.99 (0)	4.99 (0)	5.03 (0)	5.04 (0)	5.08 (0)	0.023	8.96 × 10^−148^
HDL cholesterol (mmol/L), mean ± sd	1.64 (0)	1.57 (0)	1.53 (0)	1.5 (0)	1.48 (0)	1.46 (0)	1.42 (0)	1.4 (0)	1.37 (0)	1.3 (0)	− 0.034	0.00
Triglycerides (mmol/L), mean ± sd	1.25 (0)	1.46 (0)	1.58 (0)	1.66 (0)	1.75 (0)	1.76 (0)	1.83 (0)	1.86 (0)	1.92 (0)	1.98 (0)	0.07	0.00
HbA1c (mmol/mol), mean ± sd	33.07 (0)	33.8 (0)	34.17 (0)	34.55 (0)	34.72 (0)	34.98 (0)	35.17 (0)	35.4 (0)	35.58 (0)	36.15 (0)	0.294	0.00
PXS decile, median ± sd	1 (0)	2 (0)	3 (0)	4 (0)	5 (0)	6 (0)	7 (0)	8 (0)	9 (0)	10 (0)	–	–
PRS decile, median ± sd	5 (2.9)	6 (2.87)	5 (2.86)	6 (2.86)	5 (2.86)	5 (2.86)	5 (2.88)	6 (2.88)	6 (2.87)	6 (2.86)	–	–
Males (%)	1038 (15.47)	1755 (26.41)	2400 (36.13)	2738 (41.22)	3189 (48.16)	3374 (51.18)	3752 (56.77)	4094 (62.14)	4517 (68.99)	4712 (73.19)	–	–
Family history of T2D (%)	1204 (17.94)	1337 (20.12)	1289 (19.4)	1412 (21.26)	1298 (19.6)	1310 (19.87)	1314 (19.88)	1342 (20.37)	1346 (20.56)	1322 (20.53)	–	–
High ASCVD risk (%)	197 (0.21)	514 (0.36)	1038 (0.59)	1588 (0.74)	2254 (1.04)	2767 (1.32)	3478 (1.63)	4062 (2.31)	4572 (2.5)	4853 (6.14)	–	–
Prediabetic at baseline (%)	14 (0.21)	24 (0.36)	39 (0.59)	49 (0.74)	69 (1.04)	87 (1.32)	108 (1.63)	152 (2.31)	164 (2.5)	395 (6.14)	–	–
Future incident T2D (%)	235 (3.5)	418 (6.29)	532 (8.01)	630 (9.48)	718 (10.84)	806 (12.23)	906 (13.71)	1047 (15.89)	1131 (17.28)	1472 (22.86)	–	–

Individuals who do not have T2D at baseline were binned into deciles based on their PGS (top) or PXS (bottom). The coefficient and *P* value from associations between risk score and each clinical variable are shown on the right-hand side. D1–D10: decile of risk score

We also sought to assess the risk of T2D-related complications such as heart disease. Specifically, we computed the risk of atherosclerotic cardiovascular disease (ASCVD) at baseline from the pooled cohort equation [14]. To determine high risk, we used a 7.5% risk cutoff [14]. There were 6.14% and 3.1% of individuals in the top PXS and PGS deciles, respectively, who had high predicted ASCVD risk at baseline from the pooled cohort equation.

### T2D PXS predicts longitudinal risks of other common chronic diseases

In individuals who were disease-free at baseline, T2D PXS was significantly and positively associated with the incidence of T2D and all seven chronic diseases. T2D PGS was significantly and positively associated with all diseases except atrial fibrillation (Table 3). PXS was most strongly associated with T2D (HR = 1.40 per decile increase, 95% CI 1.36–1.45, *P* = 4.26 × 10^−101^), COPD (HR = 1.34, 95% CI 1.29–1.40, *P* = 2.20 × 10^−48^), obesity (HR = 1.31, 95% CI 1.28–1.33, *P* = 3.41 × 10^−133^), and chronic liver disease (HR = 1.30, 95% CI 1.23–1.36, *P* = 7.40 × 10^−24^). PGS was most strongly associated with T2D (HR = 1.15 per decile increase, 95% CI 1.12–1.17, *P* = 4.22 × 10^−37^), chronic liver disease (HR = 1.08, 95% CI 1.04–1.12, *P* = 9.82 × 10^−5^), and obesity (HR = 1.04, 95% CI 1.0–1.06, *P* = 9.03 × 10^−7^). PXS exhibited a greater HR for PXS versus PGS for a decile increment in the score for all diseases. Similarly, PXS also exhibited a greater predictive power (Harrell’s C index) than PGS for all diseases (Table 3).

**Table 3:** Hazard ratio (HR) and sample sizes of T2D polyexposure and polygenic risks on chronic diseases

		Incident		HR (95% CI)		*P* value		C index (95% CI)	
	*N*	Diseases	T2D + disease	PXS	PGS	PXS	PGS	PXS	PGS
T2D	68,132	1182	NA	1.403 (1.360, 1.448)	1.145 (1.121, 1.169)	4.26 × 10^−101^	4.22 × 10^−37^	0.761 (0.748, 0.774)	0.725 (0.711, 0.739)
HT	51,165	4044	205	1.136 (1.118, 1.154)	1.033 (1.022, 1.045)	4.36 × 10^−57^	2.52 × 10^−09^	0.699 (0.691, 0.707)	0.690 (0.682, 0.698)
AF	67,091	1937	124	1.148 (1.136, 1.16)	1.036 (1.029, 1.043)	6.57 × 10^−149^	3.98 × 10^−22^	0.703 (0.698, 0.708)	0.691 (0.686, 0.696)
CAD	66,486	1273	89	1.141 (1.129, 1.153)	1.035 (1.028, 1.043)	4.97 × 10^−135^	2.04 × 10^−21^	0.700 (0.695, 0.705)	0.689 (0.684, 0.694)
Obesity	67,830	1974	179	1.306 (1.278, 1.334)	1.040 (1.024, 1.056)	3.41 × 10^−133^	9.03 × 10^−07^	0.711 (0.700, 0.722)	0.648 (0.636, 0.66)
CLD	67,998	360	48	1.299 (1.234, 1.366)	1.076 (1.037, 1.116)	7.40 × 10^−24^	9.82 × 10^−05^	0.713 (0.688, 0.738)	0.664 (0.637, 0.691)
CKD	67,906	969	98	1.221 (1.181, 1.264)	1.030 (1.008, 1.053)	7.58 × 10^−31^	0.00842	0.777 (0.764, 0.79)	0.759 (0.745, 0.773)
COPD	67,227	761	66	1.344 (1.292, 1.399)	1.026 (1.000, 1.052)	2.20 × 10^−48^	0.0458	0.791 (0.776, 0.806)	0.754 (0.738, 0.77)

HR of each disease is shown for each decile increase in T2D PXS and PGS after adjusting for age, sex, assessment center, and genetic principal components.

*PXS* Polyexposure risk score, *PGS* Polygenic risk score, *AF* Atrial fibrillation, *HT* Hypertension, *CAD* Coronary artery disease, *CKD* Chronic kidney disease, *COPD* Chronic obstructive pulmonary disease, *CLD* Chronic liver disease, *CI* Confidence interval

Individuals in the highest T2D PXS decile had much higher risks of other diseases (Fig. 1e, Supplementary Table 6). For example, the HR of COPD and obesity for individuals in the top T2D PXS deciles was 2.82 (95% CI 2.39–3.35, *P* = 4.00 × 10^−33^) and 2.54 (95% CI 2.24–2.87, *P* = 9.86 × 10^−50^), respectively, compared to the remaining population. In comparison, individuals in the top decile of T2D PGS had an HR of 1.36 (95% CI 1.09–1.69, *P* = 6.51 × 10^−03^) and 1.36 (95% CI 1.19–1.56, *P* = 5.75 × 10^−03^) for COPD and obesity, respectively, compared to the remaining population. For context, individuals in the top deciles (versus the bottom 9 deciles) of PXS and PGS had HR of 3.78 (95% CI 3.30–4.34, *P* = 3.27 × 10^−80^) and 2.13 (95% CI 1.83–2.48, *P* = 1.65 × 10^−22^), respectively, for incident T2D.

### PXS predicts chronic outcomes independent of established risk factors

As sensitivity analyses, we repeated our main analysis with stringent inclusion criteria on individuals who did not have diagnoses of T2D or any of the seven diseases at baseline (*N* = 49,340). The associations between risk scores and all diseases were weaker but followed a similar trend (Supplementary Table 7, Supplementary Fig. 3). We also discovered that PXS and PGS were both significantly (*P* < 0.0001) associated with the total number of incident diseases. Secondly, we repeated our main analysis while also adjusting for “established” risk factors commonly used to assess clinical risk of T2D (blood glucose, HDL cholesterol, systolic blood pressure, BMI, triglycerides, and family history of T2D) to test if PXS and/or PGS are independent of clinical risk. The associations between T2D PGS with obesity, chronic kidney disease, and COPD were no longer significant when accounting for clinical risk factors, while all associations between PXS and diseases remained statistically significant (Supplementary Table 8). We also tested the complementary and additive associations of T2D PXS and PGS by combining both risk scores into a single multivariate model. The association between PGS and COPD was no longer significant in the multivariate model, while the remaining associations remained stable, suggesting that T2D PXS and PGS have independent associations on T2D-associated diseases (Supplementary Table 9).

## Discussion

In this study, we used the novel PXSs to identify, for the first time, the shared cumulative non-genetic risks between T2D and its related chronic diseases in individuals in the UK. Individuals with environmental predispositions for T2D also have a much higher odds of certain diseases (such as cardiovascular and liver diseases). We demonstrated that PXS can identify individuals with “subclinical” T2D (individuals who are not yet diabetic, prediabetic, or obese) and risk of future T2D-related chronic diseases.

The polygenic (PGS) and polyexposure (PXS) risk scores, which summarize the additive effects of many important genetic and environmental risk factors, serve as proxies for cumulative genetic and exposure risk. Elevated PXS and PGS conferred a higher risk of T2D: Both PXS and PGS were associated with higher levels of established T2D risk factors including BMI, systolic blood pressure, blood glucose, triglycerides, and HbA1c at baseline. While we did observe a gradient in the prevalence of prediabetes as the decile of PGS and PXS increased, only a minority of individuals in the top deciles had prediabetes (i.e., 23% and 15% in the top PXS and PGS deciles, respectively). In addition to predicting incident T2D (41% increase risk of each PXS decile increase, respectively), T2D PXS is also significantly and positively associated with all other seven chronic diseases. Individuals with the highest polyexposure risks of T2D had comparable risks of COPD and obesity, with hazard ratios of 2.82 and 2.54, respectively, for the top PXS decile compared to the remaining population. We note that the predicted risk of T2D-related complications from established scoring algorithms was modest.

While prediabetes, or elevated glucose, is among the strongest risk factors for T2D [5, 15], the clinical relevance of prediabetes has been debated [16]. We demonstrate that the environmental risk of established T2D complications such as heart disease and kidney disease comes much before the onset of hyperglycemia, and, even in the absence of diagnosed diabetes. The story begins at birth with genetics and inherited risk of higher glucose levels, but continues as humans age and accrue non-genetic risk during the life course.

Individual factors that comprise the T2D PXS are also risk factors for other chronic diseases. The PXS encompasses many exposure factors, such as tobacco usage, alcohol intake, socioeconomic status, sleep patterns, and dietary factors [11], many of which have been documented to be associated with cardiometabolic disease [17–20]. However, using these factors in aggregate has not, to our knowledge, been evaluated.

Our study relies on medical records and self-reported data, which may not capture the precise time at which participants developed each disease, but rather when they were diagnosed. Furthermore, there may be errors in electronic health records or self-report. For example, others have documented underestimation of obesity in ICD codes from the UK Biobank [22]. Our study focuses on a fixed surveillance window in which the UK Biobank tracked ICD and OPCS4 codes and is not representative of lifetime risk. In our analysis, we excluded individuals with disease diagnosis at baseline to assess incident risk; thus, many participants that satisfy our inclusion criteria may be healthier than an average population. While the associations between PXS and PGS to all diseases are statistically significant, we claim the estimated sizes may be a conservative estimate and smaller due to the exclusion criteria.

In conclusion, the most common sequelae of T2D, including cardiovascular diseases, obesity, COPD, obesity and chronic kidney and liver diseases, are influenced by the same environmental exposure factors involved in incident T2D. Individuals without diabetes—or prediabetes—but have a high aggregate genetic or environmental risk of T2D also have high risks of other chronic diseases. Shared environmental risks between many chronic diseases need to be considered when assessing possible trajectories to, and from, diabetes onset.

## Research design and methods

### Phenotype classification

Diagnoses were based on self-reported data collected during interviews with a trained nurse, as well as linked hospital admission records for International Classification of Diseases (ICD) diagnostic codes and Office of Population Censuses and Surveys (OPCS-4) procedure codes recorded from hospital visits. The combined dates of diagnosis dated from 1940 to 2019.

Type 2 diabetes (T2D) cases were defined as having an ICD-10 code of E11.X, ICD-9 of 250, or having self-reported T2D in an interview (Supplementary Table 10). We excluded individuals who had a T2D diagnosis prior to age 35 years to limit the number of individuals with slow-progression autoimmune diabetes or monogenic diabetes as suggested by Klimentidis et al [23]. We also excluded individuals who did not report the age of diagnosis. Participants without diabetes were those who did not self-report T2D, had normal HbA1C levels (< 48 mmol/mol), and were not on T2D medication at the time of first assessment and did not have any documented ICD-9/10 diagnosis of T2D. We extracted T2D medications in the drug classes biguanides, sulfonylureas, meglitinides, thiazolidinediones, alpha-glucosidase inhibitors, glucose, glucagon, and insulin product from Field 2003 (Supplementary Table 11).

We examined seven other chronic diseases including atrial fibrillation (AF), coronary artery disease (CAD), hypertension (HT), obesity, chronic kidney disease (CKD), chronic obstructive pulmonary disease (COPD), and chronic liver disease (CLD). Classification of disease cases was defined by ICD-9, ICD-10, OPCS-4 codes, and self-report. Obesity was further classified by having a body mass index (BMI) greater than or equal to 30. COPD was further classified by having an FEV1/FVC ratio of less than 0.70 (Supplementary Tables 12–18). We excluded any individuals who did not report the age of diagnosis.

### Derivation of risk scores

The polygenic risk score (PGS) is a quantitative measure of an individual’s inherent genetic risk and is a weighted sum of the effects of many genetic variants. We estimated the T2D PGS using weight previously derived by Khera et al. [13] (downloaded from http://www.broadcvdi.org/informational/data). The genome-wide score consists of 6,630,149 SNPs with weight derived using LDPred, an algorithm with a linkage disequilibrium SNP-reweighting approach [24]. We used the built-in allelic scoring procedure of PLINK (--score) [25] which takes the sum of each reference allele number multiplied by the weighted coefficient across all alleles.

The polyexposure risk score (PXS) measures the non-genetic exposure risk of an individual. We estimated the T2D PXS using previously derived weights [11]. In summary, we used an iterative machine learning procedure to select 12 independent variables from a set of 111 non-genetically ascertained exposures and lifestyle factors, adjusting for age, sex, geographic location of the assessment, and genetic principal components of ancestry. The list of factors and their weights are previously described [11]. We then calculated the final polyexposure risk score by taking a weighted sum of the 12 exposure variables.

### Study population

Participants in the UK Biobank underwent genotyping with two similar arrays (UK BiLEVE Axiom Array or UK Biobank Axiom Array) consisting of over 800,000 genetic markers. Additional genotypes were imputed using the Haplotype Reference Consortium resource, the UK10K panel, and the 1000 Genomes panel, resulting in 96 million variants [26]. Information on individual background and lifestyle, cognitive and physical assessments, sociodemographic factors, and medical history was also collected for these individuals [12]. UK Biobank has ethical approval from the NHS National Research Ethics Service, and all participants provided informed consent.

### Prevalence and co-prevalence of T2D-associated diseases

We calculated the cross-sectional prevalence for seven chronic diseases in individuals who were diagnosed with T2D at any time during the surveillance period. For each individual with T2D, we randomly selected a sex- and age-matched individual who was never diagnosed with T2D during the surveillance period. We also calculated the cross-sectional prevalence for each of the seven chronic diseases in this group. The odds ratio of each disease in individuals with T2D and those without was calculated using the Fisher’s exact test.

### Clinical risks of T2D and associated diseases

We binned the polygenic (PGS) and polyexposure (PXS) risk scores into 10 deciles and calculated the average future T2D incidence, baseline BMI, systolic blood pressure, blood glucose, triglycerides, and HbA1c, and HDL cholesterols for each decile. Prediabetes was defined as having HbA1C between 39 and 48 mmol/mol. We used the *PooledCohort* package to calculate pooled cohort equations. Individuals with risk ≥ 7.5% were considered to be high risk of ASCVD [14].

### Statistical analysis

For each T2D and chronic disease pair, we retained individuals who, at the first assessment, did not have T2D or the chronic disease outcome. We then fit each of the diseases to a T2D risk score in a Cox regression model while adjusting for sex, age, forty principal components of ancestry, and assessment center. As sensitivity analyses, we also (1) included six established risk factors for T2D (blood glucose, HDL cholesterol, systolic blood pressure, BMI, triglycerides, and family history of T2D) in the Cox regression model and (2) included both PXS and PGS into a single additive model. The hazard ratio was calculated by taking the exponent of the risk score coefficient in the model. The *P* values are those of the hazard ratio of the risk scores, *i.e.,* the *P* values from testing the null hypothesis that the hazard ratio is 1. Time of event was calculated by taking the difference between the first assessment when exposures were measured to the time of diagnosis. Censoring time was derived based on an individual’s assessment center, and the date of death was derived from linked death registry data. We assessed the performance of PXS in predicting T2D by the AUC. We assessed the discrimination of PXS and PGS for each disease using the Harrell C-statistic. Cox regression was implemented by the survival package in R, and AUC was calculated using the pROC package in R.

## Supplementary Information

Below is the link to the electronic supplementary material.

Supplementary file1 (PDF 200 KB)

Supplementary file2 (XLSX 283 KB)

## Data Availability

All the data used in this study are available with the permission of the UK Biobank. PXS and PGS weights for T2D can be found at https://doi.org/10.2337/dc20-2049 and http://www.broadcvdi.org/informational/data.
