# Supplementary material for: Shared exposure liability of type 2 diabetes and other chronic conditions in the UK Biobank
Source: Acta Diabetol. 2022 Mar 29;59(6):851–60. doi: 10.1007/s00592-022-01864-5 (PMC9085680; doi:10.1007/s00592-022-01864-5)

Supplementary Figure 1: A. Cross-sectional odds ratio of each disease for individuals diagnosed with T2D versus those without T2D. B. The number of total diagnosed chronic diseases (excluding T2D) in individuals with T2D. CLD: chronic liver disease, CAD: coronary artery disease. AF: atrial fibrillation. HT: hypertension. CKD: chronic kidney disease. COPD: chronic obstructive pulmonary disease. Error bars represent 95% confidence intervals.

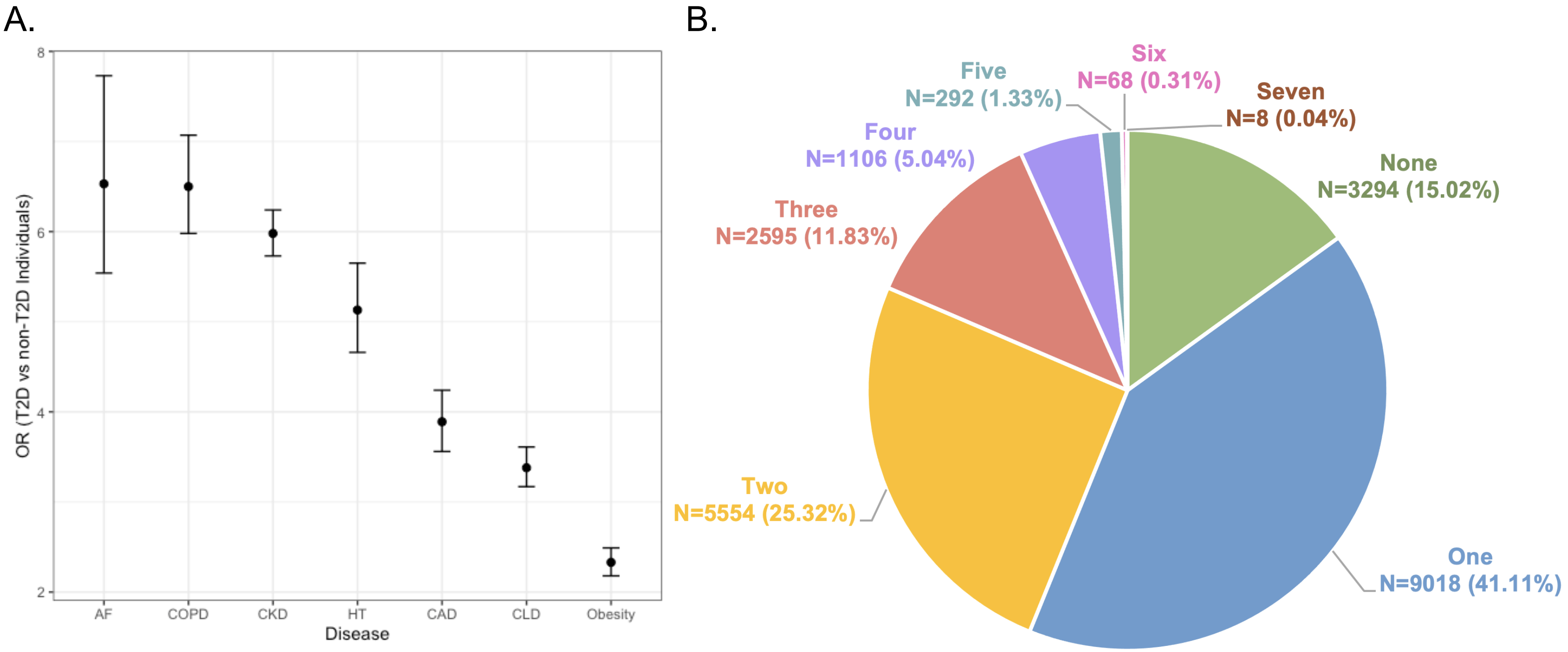

Supplementary Figure 2: Receiver operating characteristic (ROC) curve for prediction of type 2 diabetes incident cases with polyexposure risk score (PXS) alone. We estimated that the sensitivity was 0.665 and the specificity was 0.697 at the inflection point, marked by the red dotted lines.

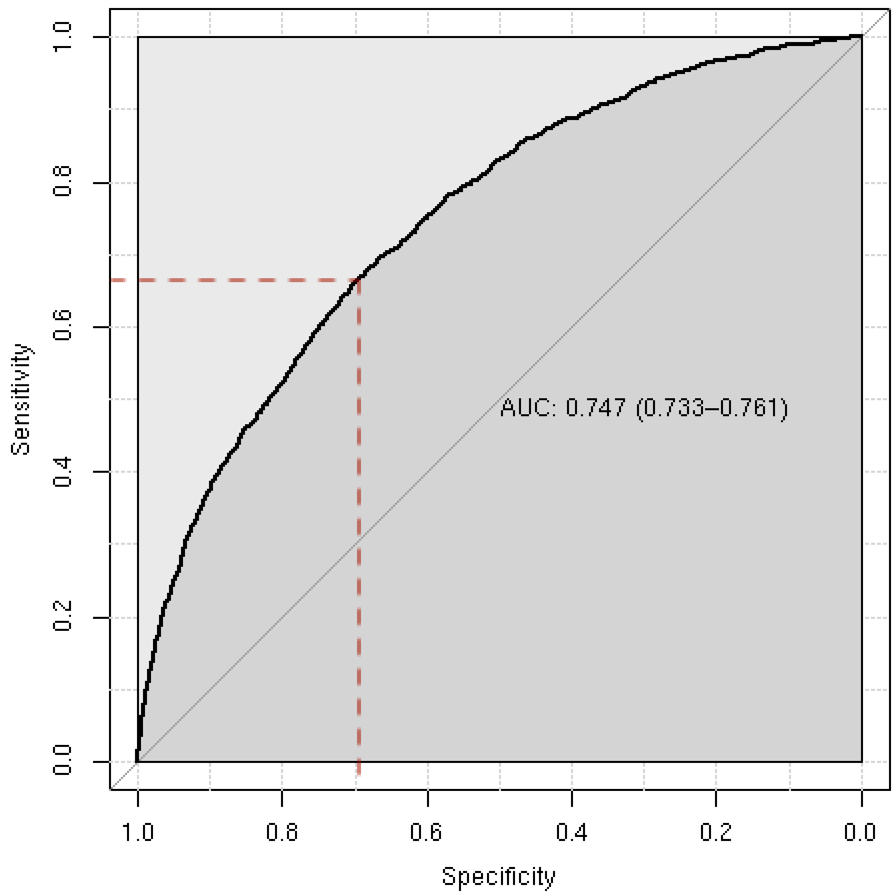

Supplementary Figure 3: Sensitivity analysis comparing hazard ratios (HR) of each disease association in the original model (x-axis) versus the model with individuals who at baseline did not have T2D or any of the other seven chronic diseases (y-axis).

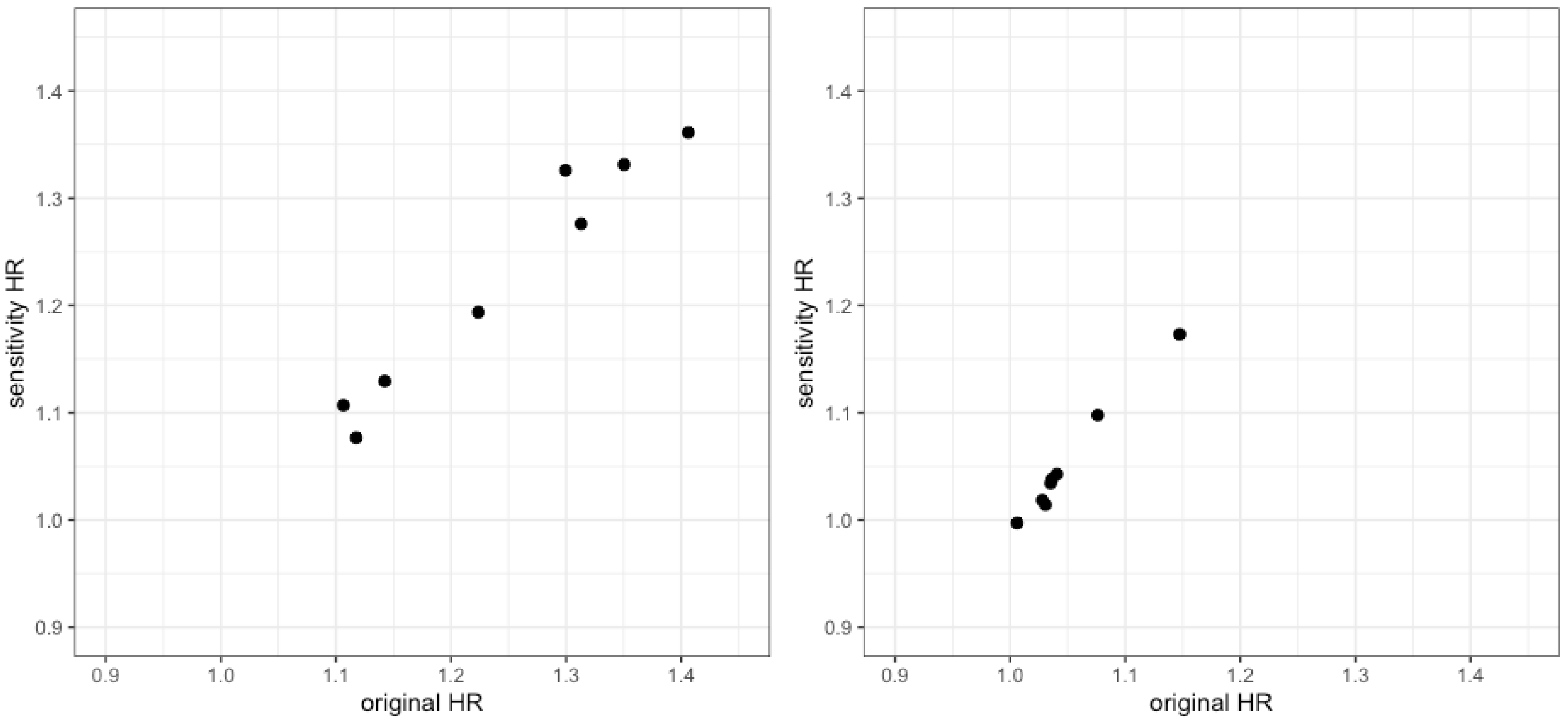

Supplement: Supplementary file 1 — Supplementary file1 (PDF 200 KB) [file 592_2022_1864_MOESM1_ESM.pdf]
